# The Association of General and Central Obesity with Major Dietary Patterns in Adult Women Living in Tehran, Iran

**Published:** 2010

**Authors:** Arezoo Rezazadeh, Bahram Rashidkhani

**Affiliations:** 1 MSc, Department of Community Nutrition, School of Nutrition and Food Technology, Shahid Beheshti University of Medical Sciences, Tehran, Iran; 2 PhD, Department of Community Nutrition, School of Nutrition and Food Technology, Shahid Beheshti University of Medical Sciences, Tehran, Iran

**Keywords:** Dietary patterns, Factor analysis, Obesity, Women, Iran

## Abstract

**BACKGROUND:**

Using dietary pattern analysis method could provide more information about nutritional etiology of chronic disease such as obesity. The aim of this study is to determine the association between major dietary patterns and general and central obesity among adult women living in Tehran.

**METHODS:**

A cross-sectional study was conducted in Tehran, Iran, with 460 women aged 20-50 years. Dietary intake in last year was collected by a semi-quantitative food frequency questionnaire. Weight, height and waist circumstance (WC) were measured with standard methods and body mass index (BMI) was calculated. General obesity was defined as BMI ≥ 30 kg/m^2^ and central obesity as WC ≥ 88 cm. Factor analysis was used for identifying major dietary patterns. The association between major dietary patterns and general and central obesity were assessed by logistic regression analysis.

**RESULTS:**

Two major dietary patterns were extracted: "Healthy" and "Unhealthy" dietary pattern. After adjusting for confounders, individuals in the highest quartile of the unhealthy dietary pattern score were more likely to be generally (OR = 7.33, 95% CI: 2.39-22.51) and centrally obese (OR = 4.99, 95% CI: 2.08-11.94); whereas, those in the upper quartile of healthy dietary pattern were less likely to be generally (OR = 0.38, 95% CI: 0.15-0.98) and centrally obese (OR = 0.33, 95% CI: 0.16-0.71).

**CONCLUSION:**

Major dietary patterns of Tehrani women had a significant association with general and central obesity. Further prospective researches are required to confirm such associations.

## Introduction

Obesity is a chronic disease that its incidence and development is affected by an interaction of demographic, socioeconomic, behavioral, cultural, physiologic and genetic factors.^1^, ^2^ Obesity is one of the most important leading causes of many chronic diseases.^3^ Obesity and central adiposity (enlarged waist circumference) prevalence has increased precipitously in Iran, especially in women, and becomes a public health concern.^4^ The nutritional transition and rapid urbanization in Iran has been associated with an overall increase in prevalence of overweight and obesity.^5^

Diet is a component of lifestyle factor that plays an important role in development of overweight and obesity.^6^, ^7^ Recently, the analysis of dietary patterns as an approach to investigating links between diet and disease has been recommended.^8^ Dietary patterns are defined by factor analysis models interrelated variables (foods) as manifestations of composite factors. These factors represent eating patterns in the study population and help to distinguish individuals according to the combination of foods they choose to eat.^9^ Furthermore, it reflects individuals' dietary behaviors and could provide more information about nutritional etiology of chronic disease such as obesity.^9^

Several studies have provided the association of major dietary patterns with general and central obesity. Nearly two-third of these studies was conducted in either Europe or North America;^10^–^20^ while, few studies have done in developing countries like Iran.^21^–^24^ Therefore, the results are representative for only a fraction of the global population. Previously, a study performed among Iranian population,^24^ has demonstrated that with factor analysis, dietary patterns were related to general and central obesity. However, this study was prone to residual confounding due to measurement error in confounders such as socioeconomic factors and family history of obesity and chronic diseases related to obesity. Furthermore, this research was performed on retired teachers aged 40-60 years old, who were similar in socioeconomic conditions. So, regarding the external validity, it tends to be necessary to investigate such associations among representative sample of women with various socioeconomic situations. The purpose of the study was to recheck the eating patterns of a reprehensive sample of Tehrani women and to assess their association with general and central obesity.

## Materials and Methods

### Participants

This cross-sectional study was conducted in a representative sample of Tehrani women aged 20–50 years selected by a stratified random-sampling method (all regions of Tehran municipality). This study was approved by the appropriate ethics committee. A sample of 516 women was invited to participate in the current study; 460 of the women agreed to proceed (the participation rate is 89%). Data were collected by trained dietitians during a structured interview, using a questionnaire. The appropriate ethics committee approved the research participants who had left > 70 items blank on the food-frequency questionnaire (FFQ) [n = 8], or participants who reported a total daily energy intake (EI) outside the range of mean ± 3 SD of energy intake [n = 11] to be excluded. Before performing the main study, a pilot study was accomplished on 50 subjects of whom none were included in the main study. The aim of the pilot study was to minimize errors and biases in collecting data.

### Assessment of dietary intake

A semi-quantitative 168-item FFQ was applied to obtain information about dietary intake over a year. The FFQ, included a list of foods (with standard serving sizes) commonly consumed by Iranians. The reported frequency for each food item was then converted to a daily intake. Portion sizes of consumed foods were converted to grams by using household measures 25. Food items included in the FFQ were grouped in to 39 predefined food groups 24. Food grouping was based on the similarity of nutrient profiles. Some individual food items were preserved either because it was inappropriate to incorporate them into a certain food group (e.g. eggs, margarine and tea) or because they were assumed to represent distinct dietary patterns (e.g. garlic or Doogh (Iranian yogurt drink)). The FFQ that was used in this study had a good relative validity and reproducibility for several nutrient intakes among Iranian (Tehrani) adults and appeared to be an acceptable tool for assessing dietary intakes in this population. Age and energy adjusted correlation coefficients between 24-h dietary recalls (repeated for 12 months) and FFQ ranged from 0.14 (vitamin A) to 0.71 (phosphorus) in men (r = 0.53) and from 0.11 (β-carotene) to 0.60 (fiber) in women (r = 0.39). Energy adjusted reproducibility coefficients varied from 0.41 (monounsaturated fat) to 0.79 (protein) in men (r = 0.59) and from 0.41 (vitamin A) to 0.74 (saturated fat) in women (r = 0.60).

### Assessment of anthropometric measures

Weight was measured to the nearest 100 g without shoes while wearing minimal clothes. Height was measured to the nearest 1 mm without shoes with shoulders in a normal position. BMI was calculated as weight in kilograms divided by height in meters squared. General obesity was defined as BMI ≥ 30 kg/m^2^. WC was measured to the nearest 1 mm by using an inelastic plastic tape measure at the narrowest point between the lowest rib and the iliac crest placed directly on the skin while the subject stood balanced on both fee. It was taken at the end of expiration. Central obesity was defined as WC ≥ 88 cm.^26^, ^27^

### Assessment of physical activity (PA)

Data was obtained using a validated self reported-based questionnaire^28^ and expressed as metabolic equivalents hour/day (METs-h/day) in which nine different MET levels were ranged on a scale from sleep/rest (0.9 METs) to high-intensity physical activities (> 6 METs). The MET-time was calculated by multiplying time spent on each activity level by the MET value of each level.

### Assessment of other variables

Additional covariate information including age, marital status, number of children, duration of residence in Tehran, socioeconomic status (university degree (yes/no), employment status (yes/no), woman income and total family income/month (USD)), current smoking, supplement use, dieting (whether or not the respondent had gone on a diet during the previous year), history of diabetes, hypertension and hyperlipidemia (whether or not diagnosed by a doctor) and family history of diabetes and obesity in first-degree relatives (parents or siblings) (yes/no), were collected by a questionnaire.

### Statistical methods

Data were analysis using SPSS software (SPSS Inc., Chicago IL. Version13). Principal component analysis was used to identify major dietary patterns based on the 39 food groups. Fourteen independent factors with eigenvalues > 1.0 were extracted. Of the derived factors, two interpretable factors were retained based on the Scree test;^29^ an orthogonal rotation procedure, the Varimax rotation, was then applied to simplify the factor structure and render it more easily interpretable. The derived factors were labeled on the basis of interpretation of the data and of the earlier literature. The factor score for each pattern was found by summing intakes of food groups weighted by factor loading,^29^ and each individual received a factor score for each identified pattern. To compare general characteristics across quartiles, analysis of variance (ANOVA) and chi-square tests were used where appropriate. The relationship between BMI and WC variables with adherence to the dietary patterns was assessed by using multiple regresion analysis in different models controlling for age, smoking, physical activity (MET-h/day) and energy intake (kJ/day) in model I, additionally for socioeconomic status (married, employed, university degree, number of children, woman income and total family income per month and duration of residence in Tehran) in model II and further for dieting, history of diabetes, hypertension and hyperlipidemia and family history of obesity and diabetes in model III. Multivariable logistic regression models were used to assess the relation of major dietary patterns with general and central obesity. Adjusted covariates were the same as above. P < 0.05 was considered significant.

## Results

Two major dietary patterns were extracted using factor analysis (Table 1): Healthy (high consumption of other vegetables, fruits, yellow vegetables, cruciferous vegetables, tomato, yogurt drink, low fat dairy products, poultry, olive, fruit juice, potato, garlic, coffee, dried fruits and legumes) and unhealthy (high in processed meats, mayonnaise, soft drinks, sweets, refined grains, snacks, industrial juice, red meats, nuts, french fries, hydrogenated fats, egg, butter, high fat dairy products, sugars and organ meats). Overall, these two factors explained 14.4% of the whole variance.

**Table 1:** Factor loading matrix for the major dietary patterns identified by using F.F.Q data among 460 Iranian women*†

	Factor 1	Factor 2
		
Food groups	(Healthy dietary pattern)	(Unhealthy dietary pattern)
Other vegetables	0.72	-
Fruits	0.67	-
Yellow vegetables	0.55	-
Cruciferous vegetables	0.49	-
Tomato	0.46	-
Yogurt drink	0.41	-
Low fat dairy products	0.40	-
Poultry	0.35	-
Olive	0.34	-
Fruit juice	0.28	-
Potato	0.25	-
Garlic	0.25	-
Coffee	0.24	-
Dried fruits	0.20	-
Legumes	0.20	-
Fish	-	-
Liquid oil	-	-
Green vegetables	-	-
Tea	-	-
Pickles	-	-
Processed meat	-	0.54
Mayonnaise	-	0.49
Soft drinks	-	0.47
Sweets	-	0.43
Refined grains	-	0.41
Snacks	-	0.40
Artificial juice	-	0.37
Red meat	-	0.35
Nuts	-	0.34
French fries	-	0.34
Hydrogenated fats	-	0.33
Egg	-	0.32
Butter	-	0.32
High fat dairy products	-	0.31
Sugars	-	0.30
Organ meats	-	0.30
Salt	-	-
Margarine	-	-
Whole wheat cereals	-	-
Processed meat	-	-

* P values < 0.20 were excluded for simplicity.

† The first factor explained 7.77% of the total variance and the second factor explained 6.63% of the total variance

Characteristics of the study participants across quartile categories of the dietary pattern scores are shown in table 2. The percentages of individuals who had university education, total family income/month > 500 USD and family history of hypertension was higher and the prevalence of central obesity was lower among subjects in the highest quartile of healthy dietary pattern score as compared with those in the lowest quartile. In contrast, subjects in the top quartile of the unhealthy dietary pattern score were younger and were less likely to have university education, history of diabetes, hypertension and hyperlipidemia, or be on a diet and they had lower duration of residence in Tehran in comparison with those in the lowest quartile. Furthermore, the prevalence of general and central obesity and the mean of BMI and WC and energy intake/day were higher (P < 0.05). Multivariate analysis of BMI and WC associated with the adherence to the two major dietary patterns are provided in table 3. BMI and WC were negatively related to healthy dietary pattern and positively associated to unhealthy one (P < 0.01). Adjustment for confounders didn't have any special effect on these results.

**Table 2:** Major characteristics of study participants according to quartiles of dietary patterns†

	Quartiles of "Healthy" dietary pattern				Quartiles of "Unealthy" dietary pattern			
	
Variables	Q1	Q2	Q3	Q4	Q1	Q2	Q3	Q4
Age (year)	32.3 (10)	32.7 (9)	34.0 (8)	35.1 (9)	28.7 (9)	22.5 (9)	21.9 (9)	21.0 (8)**
Married (%)	23.3	24.7	25.6	26.4	25.8	24.2	23.9	26.1
Number of children	2.1 (1)	2.0 (1)	1.9 (1)	2.1 (1)	2.1 (1)	2.0 (1)	1.7 (1)	1.8 (1)
Having university degree (%)	12.5	19.4	33.3	34.7**	22.2	41.7	27.8	8.3**
Employed (%)	20.7	14.4	17.4	18.2	22.7	19.8	12.8	15.2
Woman income per month (USD)	24.1 (60)	27.8 (82)	24.8 (96)	24.0 (89)	46.0 (109)	22.9 (84)	17.9 (56)	22.7 (71)
Total family income per month (USD)	409 (250)	445 (307)	512 (292)	552 (446)*	537 (537)	500 (292)	434 (287)	452 (242)
Duration of residence in Tehran (year)	25 (13)	25 (12)	24 (14)	25 (14)	29 (13)	25 (13)	23 (13)	21 (13)**
Currently smoker (%)	2.7	3.6	3.7	0.9	2.7	3.6	0.9	3.6
Supplement use (%)	23.5	20.1	27.5	28.9	24.2	30.2	22.1	23.5
Dieting (%)	3.6	8.1	6.4	5.5	14.5	3.6	2.8	2.7**
Physical activity†† (MET)	25 (6)	25 (6)	26 (7)	26 (7)	25 (7)	26 (6)	24 (7)	26 (7)
History of diabetes (%)	5.4	5.4	5.5	6.4	10.9	6.3	3.6	2.8*
History of hypertension (%)	4.5	5.4	6.4	13.6*	15.5	6.3	4.5	4.0**
History of hyperlipidemia (%)	9.0	9.9	11.9	18.2	21.8	12.6	8.3	6.3**
Family history of obesity (%)	35.0	39.5	33.1	39.1	36.4	36.8	38.6	35.0
Family history of diabetes (%)	15.3	14.4	18.3	20.9	23.6	18.9	11.0	15.3
Body mass index (Kg/m^2^)	26 (3)	26 (3)	25 (3)	25 (3)	24 (3)	25 (3)	25 (3)	27 (4)**
Waist circumference (cm)	85.2 (8)	86.5 (9)	82.7 (7)	84.2 (8)	82.9 (7)	83.4 (8)	84.7 (8)	87.7 (9)**
Generally obese (%)	17.1	12.5	11.0	11.8	9.1	8.1	9.2	27.0**
Centrally obese (%)	33.4	32.3	21.1	20.0*	15.5	23.2	25.9	42.3**

† Data are presented as n (%) or mean (standard deviation)

†† MET: metabolic equivalent task; 1 MET = energy expenditure of sitting quietly or approximately 1 kcal per kg body weight per hour

* Means are significantly different: P < 0.05;

** Means are significantly different: P < 0.01.

**Table 3:** The assosiation of BMI and waist circumference(WC) with the adherence to major dietary patterns among 460 Iranian women aged 20–50 yaers old

	BMI		WC	
	
	b†	CI†† (95%)	b	CI (95%)
**Healthy dietary pattern**				
Crude	- 0.40*	-0.75 to -0.05	- 0.78**	-1.58 to 0.01
Model I‡	- 0.80**	-1.15 to -0.44	- 1.81**	- 2.62 to -0.01
Model II‡‡	- 0.74**	-1.14 to -0.33	- 1.60**	-2.52 to -0.69
Model III§	- 0.73**	-1.13 to -0.32	- 1.60**	-2.51 to -0.69
**Unhealthy dietary pattern**				
Crude††	1.09**	0.76 to 1.42	1.70**	0.91 to 2.49
Model I‡	1.65**	1.21 to 2.09	2.65**	1.63 to 3.68
Model II‡‡	1.74**	1.25 to 2.23	2.59**	1.45 to 3.73
Model III§	1.75**	1.26 to 2.23	2.61**	1.48 to 3.74

† b = Regression coefficient

†† CI = Confidence interval

‡ Model I: adjusted for age, smoking, physical activity and energy intake (kJ/day)

‡‡ Model II: Additionally adjusted for socioeconomic status (marital status, number of children, university degree, employment status, woman income and total family income per month and duration of residence in Tehran)

§ Model III: Further adjusted for dieting, history of diabetes, hypertension and hyperlipidemia and family history of obesity and diabetes

* Means are significantly different: P < 0.05;

** Means are significantly different: P < 0.01

In Table 4, OR for general and central obesity across quartile of dietary pattern scores are presented. After adjusting for all confounders, individuals in the highest quartile of the unhealthy dietary pattern score were more likely to be generally (OR = 7.33, 95% CI: 2.39-22.51) and centrally obese (OR = 4.99, 95% CI: 2.08-11.94); whereas, those in the upper quartile of healthy dietary pattern were less likely to be generally (OR = 0.38, 95% CI: 0.15-0.98) and centrally obese (OR = 0.33, 95% CI: 0.16-0.71).

**Table 4:** Multivariate adjusted odds ratios for general obesity and central adiposity across quartiles of dietary pattern scores among 460 Iranian women aged 20–50 years old†

	Quartiles of healthy pattern score				Quartiles of unhealthy pattern score			
		
	1	2	3	4	1	2	3	4
**General obesity**††								
Crude	1.00	0.75 (0.36-1.57)	0.59 (0.27-1.30)	0.64 (0.30-1.38)	1.00	0.88 (0.34-2.26)	1.01 (0.40-2.52)	3.7 (1.70-8.02)**
Model I‡	1.00	0.79 (0.37-1.69)	0.49 (0.22-1.10)	0.40 (0.17-0.92)*	1.00	1.25 (0.45-3.32)	1.68 (0.60-4.66)	7.14 (2.60-19.52)**
Model II‡‡	1.00	0.76 (0.33-1.75)	0.54 (0.22-1.30)	0.42 (0.16-1.04)*	1.00	1.35 (0.48-3.74)	1.75 (0.59-5.19)	7.41 (2.45-22.45)**
Model III§	1.00	0.76 (0.33-1.77)	0.53 (0.21-1.31)	0.38 (0.15-0.98)*	1.00	1.25 (0.44-3.52)	1.66 (0.54-5.03)	7.33 (2.39-22.51)**
**Central obesity**§§								
Crude	1.00	1.04 (0.59-1.82)	0.55 (0.30-1.02)	0.50 (0.28-0.96)**	1.00	1.84 (0.94-3.60)	1.71 (0.86-3.38)	4.01 (2.11-7.61)**
Model I‡	1.00	1.08 (0.60-1.93)	0.44 (0.33-0.83)	0.31 (0.16-0.62)**	1.00	2.43 (1.21-4.91)	2.51 (1.17-5.38)	6.40 (2.84-14.41)**
Model II‡‡	1.00	1.05 (0.55-2.02)	0.45 (0.22-0.91)	0.35 (0.16-0.74)**	1.00	2.36 (1.12-5.00)	2.27 (1.03-5.01)	4.92 (2.08-11.63)**
Model III§	1.00	1.02 (0.53-1.97)	0.45 (0.22-1.92)	0.33 (0.16-0.71)**	1.00	2.40 (1.12-5.13)	2.32 (1.03-5.23)	4.99 (2.08-11.94)**

† Values are OR (95% CI)

†† General obesity: BMI ≥ 30 kg/m2

‡ Model I: Adjusted for age, smoking, physical activity and energy intake

‡‡ Model II: Additionally adjusted for socioeconomic status (marital status, number of children, university degree, employment status, woman income and total family income per month and duration of residence in Tehran)

§ Model III: Further adjusted for dieting, history of diabetes, hypertension and hyperlipidemia and family history of obesity and diabetes

§§ Central obesity: WC ≥ 88 cm.

* P-trend is significantly different: P < 0.05;

** P-trend is significantly different: P < 0.01

## Discussion

Two major dietary patterns were identified in this population that named "Healthy" and "Unhealthy". The healthy dietary pattern was inversely associated with the risk of general and central obesity; while, the unhealthy dietary pattern was positively related.

The strengths of this study are the population-based design and high participation rate (more than 90 percent). Another strength point was adjustment of various confounders specially dieting and history of diabetes, hypertension and hyperlipidemia. Presence of these factors can be affected on usual dietary patterns; because, people inclined to changes in dietary habits and to adopt some healthier and more protective diets following advice from their doctors in order to control their weight or disease;^3^, ^30^ furthermore, results were adjusted for the presence of cancer or CVD without changes in the estimations. In the other hand, adjustment of family history of diabetes and obesity can relatively control the effect of genetic on obesity. Finally, we adjusted the effect of demographic and socioeconomic factors. Also, physical activity was adjusted assuming that weight gain in adults depends on the balance between the expenditure and energy intake.^21^ Dietary patterns are highly influenced by socioeconomic factors. In our previous research,^31^ multivariate analysis showed that dietary patterns of Tehrani females were related to age, university degree, smoking, ethnicity, income, owning house and duration of residence in Tehran.

The dietary patterns identified in this study were similar to those found in a previous study done in Iran, using factor analysis of dietary intake. Esmaillzadeh and Azadbakht,^24^ identified 3 major dietary patterns in Tehrani female teachers aged 40-60 years, named "healthy" (high in fruits, vegetables, poultry, legumes, tea and whole grains), "western" (high in refined grains, red meats, butter, processed meats, high-fat dairy products, sweets and desserts, potatoes, eggs and hydrogenated fats), and "Iranian" (high in refined grains, potato, tea, whole grains, hydrogenated fats, legumes, and broth) dietary patterns, which healthy and western patterns were similar to identified dietary patterns in our study.

In the other hand, the healthy and unhealthy patterns of current study were consistent with "prudent" and "western" dietary patterns in Health Professionals' Follow-up Study,^32^ and "healthy" and "western" patterns in Swedish^33^ and American^34^ women. Likewise, they were comparable with "Spanish-Mediterranean" (as a healthy pattern) and "western" pattern in Spanish men and women participating in Spanish SUN project (Seguimiento Universidad de Navarra).^3^ However, comparison and interpretation of dietary patterns derived from different studies should be done with caution due to geographical and cultural as well as methodological variations.^35^

In this study, the relationship between healthy and unhealthy dietary patterns and risk of general and central adiposity were similar to findings of the study performed among 40-60 years Tehrani female teachers.^24^ In that study, women in the upper quintile of the healthy pattern were less likely to be generally and centrally obese, whereas, those in the upper category of western pattern had greater odds. Furthermore, a previously study in an Asian country (Japan) presented consistent results.^23^ In this study, the "Healthy" pattern (high in vegetables, mushrooms, seaweeds, potatoes, fish and shellfish, soy products, processed fish, fruit and salted vegetables) was related to lower risk of overweight and obesity and the "western" pattern, (high intakes of meats, fats and oils, seasonings, processed meats and eggs) were associated with an increased risk of BMI ≥ 25.

The inverse association of healthy pattern with risk of general and central obesity was similar to reported findings in American^26^, ^28^ and European^23^ countries. In other studies, the inverse relation of BMI and weight gain with "whole grains, fruits and vegetables" pattern were reported in men^36^ and women^16^ participated in the Nurses' Health Study. Also, a "low-fat dairy, grains, and fruit" pattern in American women had an inverse association with annual changes in BMI and WC.^10^, ^11^

Otherwise, the unhealthy dietary pattern was positively related to increased risk of general and central adiposity. In other studies, the western pattern (which is similar to our unhealthy pattern) showed the same results.^13^, ^16^, ^17^, ^20^, ^23^ Also, various dietary patterns high in "meat and pasta"^30^ and "refined grains and hotdogs"^37^ were positively related to BMI and a pattern high in "rice"^18^ was associated to general and central adiposity. Western content of this dietary pattern reflects the effect of nutrition transition on obesity in Iranian women.

The protective effect of healthy dietary pattern may be due to the effect of food with high fiber and complex carbohydrates, low glycemic index and low energy density such as vegetables, fruits and legumes^38^, ^39^ and low fat consumption on appetite and food intake.^40^ While, the positive relation between unhealthy pattern and these conditions can be explained by over consumption of higher glycemic index carbohydrates (refined grains, sugars and sweets) which cause higher glycemic responses and increased fat synthesis and fat accumulation.^21^ Furthermore, these kinds of carbohydrates increase hunger and promote overeating. In the other hand, higher fat intake, which is seen in unhealthy dietary pattern, has been considered a risk factor for weight gain 41. Some points should be considered in interpreting these findings. First, due to the cross-sectional design of the study, one cannot infer causality. Therefore, our findings need to be confirmed in future prospective studies. Second, limitations of FFQ for assessing dietary intakes should be taken into account (such as measurement errors inherent to the use of a FFQ for dietary assessment include underreporting or overreporting of general food intake, selective underreporting or overreporting of intakes of certain foods, or both).^42^ Third, several subjective or arbitrary decisions in the use of factor analysis need to be considered; the investigator is forced to prespecify the number of factors. Although eigen values, Scree plots, and interpretability are used to guide the investigator in determining the best factor solution, ultimately such a decision is subjective.^11^, ^43^

## Conclusion

Our findings suggest the protective effect of healthy dietary pattern, characterized by high consumption of vegetables fruits, low fat dairy products and legumes, against general and central obesity, while unhealthy dietary pattern with high consumption of processed and red meats, fatty foods, refined grains and sugars is associated with higher risk of these kinds of adiposities. Further prospective research studies are needed to approve these relations.

## References

[1] Yang EJ, Kerver JM, Song WO (2005). Dietary patterns of Korean Americans described by factor analysis. J Am Coll Nutr.

[2] National Institutes of Health (NIH), National Heart, Lung, Blood Institute (NHLBI) (1998). Clinical guidelines on the identification, evaluation, and treatment of overweight and obesity in adults.

[3] Sanchez-Villegas A, Delgado-Rodriguez M, Martinez-Gonzalez MA, Irala-Estevez J (2003). Gender, age, socio-demographic and lifestyle factors associated with major dietary patterns in the Spanish project SUN (Seguimiento Universidad de Navarra). Eur J Clin Nutr.

[4] Azizi F, Azadbakht L, Mirmiran P (2005). Trends in overweight, obesity and central fat accumulation among Tehranian adults between 1998-1999 and 2001-2002: Tehran lipid and glucose study. Ann Nutr Metab.

[5] Ghassemi H, Harrison G, Mohammad K (2002). An accelerated nutrition transition in Iran. Public Health Nutr.

[6] Azadbakht L, Mirmiran P, Shiva N, Azizi F (2005). General obesity and central adiposity in a representative sample of Tehranian adults: prevalence and determinants. Int J Vitam Nutr Res.

[7] Azadbakht L, Esmaillzadeh A (2008). Dietary and non-dietary determinants of central adiposity among Tehrani women. Public Health Nutr.

[8] Kant AK (2004). Dietary patterns and health outcomes. J Am Diet Assoc.

[9] Hu FB (2002). Dietary pattern analysis: a new direction in nutritional epidemiology. Curr Opin Lipidol.

[10] Newby PK, Muller D, Hallfrisch J, Qiao N, Andres R, Tucker KL (2003). Dietary patterns and changes in body mass index and waist circumference in adults. Am J Clin Nutr.

[11] Newby PK, Muller D, Hallfrisch J, Andres R, Tucker KL (2004). Food patterns measured by factor analysis and anthropometric changes in adults. Am J Clin Nutr.

[12] Newby PK, Weismayer C, Akesson A, Tucker KL, Wolk A (2006). Longitudinal changes in food patterns predict changes in weight and body mass index and the effects are greatest in obese women. J Nutr.

[13] Maskarinec G, Novotny R, Tasaki K (2000). Dietary patterns are associated with body mass index in multiethnic women. J Nutr.

[14] Quatromoni PA, Copenhafer DL, D'Agostino RB, Millen BE (2002). Dietary patterns predict the development of overweight in women: the framingham nutrition studies. J Am Diet Assoc.

[15] Mendez MA, Popkin BM, Jakszyn P, Berenguer A, Tormo MJ, Sanchez MJ (2006). Adherence to a mediterranean diet is associated with reduced 3-year incidence of obesity. J Nutr.

[16] Schulze MB, Fung TT, Manson JE, Willett WC, Hu FB (2006). Dietary patterns and changes in body weight in women. Obesity (Silver Spring).

[17] Schulz M, Kroke A, Liese AD, Hoffmann K, Bergmann MM, Boeing H (2002). Food groups as predictors for short-term weight changes in men and women of the EPIC-Potsdam cohort. J Nutr.

[18] Lin H, Bermudez OI, Tucker KL (2003). Dietary patterns of Hispanic elders are associated with acculturation and obesity. J Nutr.

[19] McNaughton SA, Mishra GD, Stephen AM, Wadsworth ME (2007). Dietary patterns throughout adult life are associated with body mass index, waist circumference, blood pressure, and red cell folate. J Nutr.

[20] Murtaugh MA, Herrick JS, Sweeney C, Baumgartner KB, Guiliano AR, Byers T (2007). Diet composition and risk of overweight and obesity in women living in the southwestern United States. J Am Diet Assoc.

[21] Sichieri R (2002). Dietary patterns and their associations with obesity in the Brazilian city of Rio de Janeiro. Obes Res.

[22] Shimazu T, Kuriyama S, Hozawa A, Ohmori K, Sato Y, Nakaya N (2007). Dietary patterns and cardiovascular disease mortality in Japan: a prospective cohort study. Int J Epidemiol.

[23] Okubo H, Sasaki S, Murakami K, Kim MK, Takahashi Y, Hosoi Y (2008). Three major dietary patterns are all independently related to the risk of obesity among 3760 Japanese women aged 18–20 years. Int J Obes (Lond).

[24] Esmaillzadeh A, Azadbakht L (2008). Major dietary patterns in relation to general obesity and central adiposity among Iranian women. J Nutr.

[25] Lopez-Garsia E, Schulze MB, Fung TT, Manson JE, Hu FB, Rifai N (2004). Major dietary patterns are related to plasma concentrations of markers of inflammation and endothelial dysfunction. American Journal of Clinical Nutrition.

[26] Lohman TG, Roche AF, Martorell R (1988). Anthropometric standardization reference manual.

[27] Wang J, Thornton JC, Bari S, Williamson B, Gallagher D, Heymsfield SB (2003). Comparisons of waist circumferences measured at 4 sites. Am J Clin Nutr.

[28] Aadahl M, Jorgensen T (2003). Validation of a new self-report instrument for measuring physical activity. Med Sci Sports Exerc.

[29] Kim JO, Mueller CW (1978). Factor analysis: statistical methods and practical issues.

[30] Pala V, Sieri S, Masala G, Palli D, Panico S, Vineis P (2006). Associations between dietary pattern and lifestyle, anthropometry and other health indicators in the elderly participants of the EPIC-Italy cohort. Nutr Metab Cardiovasc Dis.

[31] Rezazadeh A, Rashidkhani B, Omidvar N (2010). Association of major dietary patterns with socioeconomic and lifestyle factors of adult women living in Tehran, Iran. Nutrition.

[32] Hu FB, Rimm E, Smith-Warner SA, Feskanich D, Stampfer MJ, Ascherio A (1999). Reproducibility and validity of dietary patterns assessed with a food-frequency questionnaire. Am J Clin Nutr.

[33] Khani BR, Ye W, Terry P, Wolk A (2004). Reproducibility and validity of major dietary patterns among Swedish women assessed with a food-frequency questionnaire. J Nutr.

[34] Slattery ML, Boucher KM, Caan BJ, Potter JD, Ma KN (1998). Eating patterns and risk of colon cancer. Am J Epidemiol.

[35] Fung TT, Willett WC, Stampfer MJ, Manson JE, Hu FB (2001). Dietary patterns and the risk of coronary heart disease in women. Arch Intern Med.

[36] Hu FB, Rimm EB, Stampfer MJ, Ascherio A, Spiegelman D, Willett WC (2000). Prospective study of major dietary patterns and risk of coronary heart disease in men. Am J Clin Nutr.

[37] Schulze MB, Hoffmann K, Kroke A, Boeing H (2001). Dietary patterns and their association with food and nutrient intake in the European Prospective Investigation into Cancer and Nutrition (EPIC)-Potsdam study. Br J Nutr.

[38] Jenkins DJ, Kendall CW, Augustin LS, Franceschi S, Hamidi M, Marchie A (2002). Glycemic index: overview of implications in health and disease. Am J Clin Nutr.

[39] Bell EA, Castellanos VH, Pelkman CL, Thorwart ML, Rolls BJ (1998). Energy density of foods affects energy intake in normal-weight women. Am J Clin Nutr.

[40] Kimm SY (1995). The role of dietary fiber in the development and treatment of childhood obesity. Pediatrics.

[41] Roberts SB (2000). High-glycemic index foods, hunger, and obesity: is there a connection?. Nutr Rev.

[42] Kerver JM, Yang EJ, Bianchi L, Song WO (2003). Dietary patterns associated with risk factors for cardiovascular disease in healthy US adults. Am J Clin Nutr.

[43] Martinez ME, Marshall JR, Sechrest L (1998). Invited commentary: factor analysis and the search for objectivity. Am J Epidemiol.

